# Feasibility of Using Synthetic Aperture Radar to Aid UAV Navigation

**DOI:** 10.3390/s150818334

**Published:** 2015-07-28

**Authors:** Davide O. Nitti, Fabio Bovenga, Maria T. Chiaradia, Mario Greco, Gianpaolo Pinelli

**Affiliations:** 1 Geophysical Applications Processing s.r.l., Via Amendola 173, 70126 Bari, Italy; E-Mail: davide.nitti@gapsrl.eu; 2 National Research Council of Italy, ISSIA institute, Via Amendola 173, 70126 Bari, Italy; 3 Dipartimento di Fisica “M. Merlin”, Politecnico di Bari, Via Amendola 173, 70126 Bari, Italy; E-Mail: chiaradia@ba.infn.it; 4 IDS—Ingegneria Dei Sistemi S.p.A., Via Enrica Calabresi 24, 56121 Pisa, Italy; E-Mails: m.greco@idscorporation.com (M.G.); g.pinelli@idscorporation.com (G.P.)

**Keywords:** UAV, navigation, Geo-referencing, SAR, interferometry, ATR, feasibility

## Abstract

This study explores the potential of Synthetic Aperture Radar (SAR) to aid Unmanned Aerial Vehicle (UAV) navigation when Inertial Navigation System (INS) measurements are not accurate enough to eliminate drifts from a planned trajectory. This problem can affect medium-altitude long-endurance (MALE) UAV class, which permits heavy and wide payloads (as required by SAR) and flights for thousands of kilometres accumulating large drifts. The basic idea is to infer position and attitude of an aerial platform by inspecting both amplitude and phase of SAR images acquired onboard. For the amplitude-based approach, the system navigation corrections are obtained by matching the actual coordinates of ground landmarks with those automatically extracted from the SAR image. When the use of SAR amplitude is unfeasible, the phase content can be exploited through SAR interferometry by using a reference Digital Terrain Model (DTM). A feasibility analysis was carried out to derive system requirements by exploring both radiometric and geometric parameters of the acquisition setting. We showed that MALE UAV, specific commercial navigation sensors and SAR systems, typical landmark position accuracy and classes, and available DTMs lead to estimate UAV coordinates with errors bounded within ±12 m, thus making feasible the proposed SAR-based backup system.

## 1. Introduction

This study is devoted to explore the potentials of synthetic aperture radar (SAR) and Interferometric SAR (InSAR) to aid unmanned aerial vehicle (UAV) navigation. Over the past decades, UAVs have been increasingly used for a wide range of both civilian and military applications, such as reconnaissance, surveillance and security, terrain mapping and geophysical exploration. The feasible use of a UAV for a certain application relies on the accuracy and robustness of the navigation system. The navigation of a UAV is controlled by the inertial navigation system (INS), which exploits different sensors such as inertial measurement unit (IMU), radar altimeter (RALT) and global positioning system (GPS) receiver. These sensors allow the UAV to measure the status vectors of the aircraft (position, velocity, acceleration, Euler attitude angle and rates) needed to infer the actual trajectory with enhanced accuracy. Thus, the INS defines the commands needed to change the status vectors in order to guide the aircraft along the mission reference trajectory, or can be used to provide geo-referenced products acquired onboard for ground control point free applications [1].

The INS performance and (consequently) the navigation accuracy depend on the performance of such sensors and, in particular, on the IMU. It is made of three gyroscopes and three accelerometers, which are used to calculate attitude, absolute acceleration and velocity of the aircraft [2]. One of the main drawbacks of IMU is the rapid growth of systematic errors as bias and drift, which have to be compensated by using the absolute positions measured by the GPS [3]. However, due to the low power of the ranging signals, the received GPS signal is easily corrupted or completely obscured by either intentional (e.g., jamming) or unintentional interferences. Moreover, along the UAV trajectory the GPS signal can be also absent. When GPS data are not reliable or absent, the correction of the aircraft trajectory through the IMU is consequently unfeasible. 

This problem can affect in particular medium-altitude long-endurance (MALE) UAV, which has to fly for thousands of kilometers before reaching its target. Cumulative drift (up to hundreds of meters) during a long endurance flight can lead the UAV to miss the planned target with catastrophic consequences on the mission [4]. Thus, future guidance systems for UAV autonomous missions have challenging requirements for high reliability and integrity. To fully meet these new requirements, the following capacities need to be radically improved: enhanced tolerance to GPS denial/jamming, higher IMU performance, and reduced INS drift. 

The aim of this work is to assess the feasibility of using SAR to aid a standard navigation system when IMU measurements are not accurate enough to eliminate drifts from a planned trajectory. Examples of SAR systems mounted onboard UAVs already exist (e.g*.*, [5,6]) but not devoted to aid the platform navigation. The basic idea is to infer position and attitude of an aerial platform by inspecting both amplitude and phase of SAR images provided by a SAR system onboard the platform. SAR data provide information on the electromagnetic microwave backscatter characteristics of the Earth surface with day/night and all-weather capability, thanks to the active nature of radar sensors [7]. Moreover, the SAR imaging is able to illuminate a wide area on the ground (up to several square kilometers) from a long distance (up to tens of kilometers) by ensuring high spatial resolution (meters or less depending on the bandwidth). These characteristics are advantageous with respect to other sensors (e.g., optical) and make suitable the proposed backup navigation system. 

**Figure 1:**
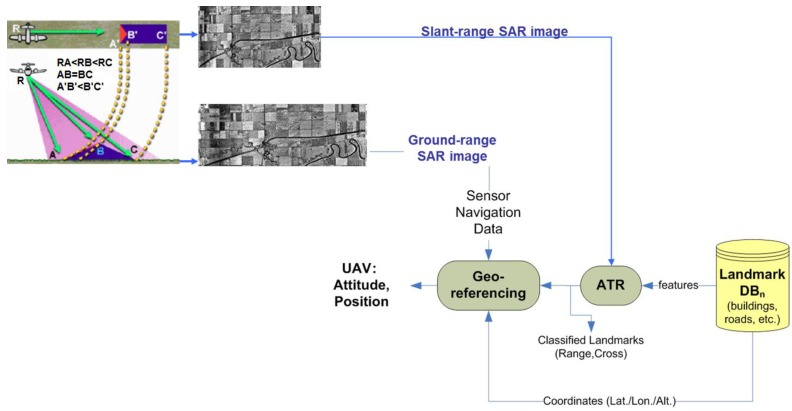
Whole ATR and SAR based geo-referencing concept: processing-flow.

In case of the amplitude-based approach, the system navigation correction can be based on a comparison between processed SAR images and a terrain landmark Data Base (DB), which contains the geographic coordinates of expected (during the UAV mission) ground landmarks (*i.e.*, conspicuous objects on land that unequivocally mark a locality). Let us assume that the scene acquired by the SAR onboard the platform is populated by terrain landmarks (e.g., roads, buildings), which is quite likely in several operating scenarios (e.g., rural, industrial, suburban). The image coordinates (range/cross-range) of the expected landmarks can be automatically extracted by an Automatic Target detection and Recognition (ATR) algorithm for SAR images [8]. Then, the coordinates of an image landmark have to be correlated with the geographic coordinates of the corresponding landmark in the mission DB. Once a match is found, a mathematical relation between range/cross-range landmark coordinates and landmark geographic coordinates can be found and exploited to retrieve aircraft position. The whole ATR and SAR based geo-referencing block is depicted in the processing flow in Figure 1. The onboard SAR acquires an image under a certain viewing geometry; terrain landmarks in the focused image are extracted by an ATR processing chain that is fed by a landmark DB; mission planned landmarks are recognized by the ATR chain; the ATR block provides the coordinates of each landmark both in the SAR coordinate system and in the DB inertial coordinate system; local and inertial coordinates of each landmark, as well the initial aircraft attitude/position measurements (from the INS) are exploited by the geo-referencing algorithm; the final output is the position of the aircraft in the inertial coordinate system. 

When the use of SAR amplitude is unfeasible, the phase content of the SAR image is exploited through a real time InSAR system mounted onboard the platform. By synthetically coupling SAR images acquired by different positions [9], the InSAR system provides information about the radiation path delay between two acquisitions, which includes the topography, and any difference occurred between the two passes on the ground surface as well as in the atmospheric refractivity profile. The InSAR phase derived by using a single-pass interferometry system avoids contributions from both ground deformation and atmosphere, and is related only to the aircraft position and to the topography. Therefore, by using both approximated position and attitude values of the platform, and a reference DTM, it is possible to generate a synthetic InSAR phase model to be compared with respect to (w.r.t.) that derived by processing the InSAR images. The geometrical transformation needed to match these two terrain models depends on the difference between the actual values of position and attitude, and those derived by the instruments available onboard. Hence, this matching provides a feedback to be used for adjusting position and attitude when a lack of GPS signal leads to unreliable IMU data. 

The goal of the paper is to propose an advance in the integration of radar imaging into UAV navigation systems and to prove the technological feasibility of the proposed integrated system. Nowadays, medium-altitude long-endurance (MALE) UAV class [10] permits heavy (in the order of hundreds of kilograms) and wide (in the order of few square meters) payloads to be carried onboard, during a long mission (in the order of few thousands of kilometers), thus making feasible the use of SAR systems, which are more demanding than other sensors in terms of weight and space (the InSAR configuration in particular). Some examples of MALE UAVs are Predator B, Global Hawk and Gray Eagle, which are already equipped with SAR systems and can be also equipped with InSAR systems. The paper provides a feasibility analysis performed by simulating realistic scenarios according to the characteristics of commercial navigation sensors and SAR systems, typical landmark position accuracy and classes, and available DTMs. 

Section 2 describes the amplitude-based approach, and presents a feasibility analysis that provides requirements for aerial platform class, onboard navigations sensors, the SAR system and acquisition geometry, landmark DB and mission planning. In Section 3, we first introduce the InSAR configuration with preliminary consideration concerning the expected InSAR phase quality. Then, we evaluate the InSAR sensitivity to changes on aircraft position and attitude. Requirements for the DTM are also provided. Finally, in the conclusions section, we resume the limits of applicability and provide indications concerning the system parameters. 

## 2. SAR Amplitude Exploitation

### 2.1. Proposed Approach

A concept scheme of the proposed SAR amplitude-based approach is depicted in Figure 1. Novel and reliable system navigation correction is necessary and can be based on the best matching between planned (latitude/longitude/altitude) and automatically extracted (azimuth/slant-range) landmark (*i.e.*, a conspicuous object on land that unequivocally marks a locality) coordinates as a function of aircraft state variables. The automatic extraction step is performed by an ATR chain, which is able to automatically extract from the SAR image the landmarks (e.g., buildings, crossroads) in the mission DB, which contains several features (e.g., landmark position, size). Finally, both extracted radar coordinates (those obtained under the actual SAR viewing geometry) and the world coordinates (those in the mission DB) of the same landmark are used for retrieving aircraft state variables. For simplicity of representation, a landmark can be modeled as a set of Ground Control Points (GCPs), e.g*.*, a cross-road landmark can be represented by the coordinates of four GCPs.

As a GPS denial occurs, the backup system triggers the SAR sensor for the next Way Point (WP) and controls the radar beam steering onto the expected terrain landmark previously stored in the onboard DB. The beam steering must ensure enough time on the landmark to gather the echo returns needed to focus the raw data image. It is worth noting that the proposed approach and the feasibility analysis here presented are more focused on the UAV platform, SAR system and navigation sensors than on the image processing chain (SAR data autofocusing [5,6], ATR chain), which is beyond the scope of this paper. 

### 2.2. Basic Concepts and Geo-Referencing Procedure

**Figure 2:**
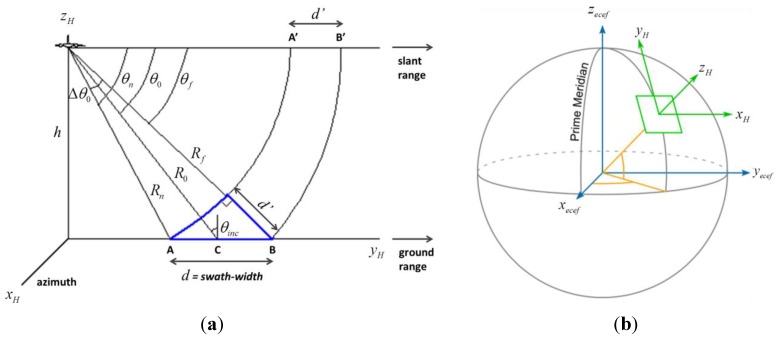
(**a**) Airborne side looking SAR geometry; (**b**) Basic flat-Earth geometry.

The whole (SAR amplitude-based) geo-referencing procedure is depicted in the processing-flow in Figure 1. It can be noted that viewing geometry and SAR geometrical distortions have to be considered in order to derive a meaningful geo-referencing algorithm based on the recognition of landmarks in SAR images. A basic viewing geometry of an SAR is shown in Figure 2a [8]: a platform flying with a given velocity at altitude *h* carries a *side-looking* radar antenna that illuminates the Earth’s surface with pulses of electromagnetic radiation. The direction of travel of the platform is known as the *azimuth* direction, while the distance from the radar track is measured in the *slant range* direction. The *ground range* and its dependence on the angle *θ* is also depicted. Note that in Figure 2b we assumed the flat Earth approximation, which can be considered valid for the airborne case, even for long-range systems [11]. Before presenting the proposed procedure, we introduce some quantities that will be used in the following. A complete list can be found in Table 1. 

*Inertial system of coordinates*: classically, it is the Cartesian system of coordinates. In the geodetic literature, an inertial system is an Earth-Centred-Earth-Fixed (ECEF) frame where the origin is at the Earth’s centre of mass, the (*x_ecef_*, *y_ecef_*) plane coincides with the equatorial plane, and the (*x_ecef_*, *z_ecef_*) plane contains the first meridian) [12]. 

*Local flat Earth:* position of a point $p_{H}={\left[x_{H},y_{H},z_{H}\right]}^{t}$ can be computed from a geodetic latitude-longitude-altitude frame or an ECEF frame by assuming that the flat Earth *z_H_*-axis is normal to the Earth only at the initial geodetic coordinates [13]. For our application, the flat Earth model can be assumed if *h* << *R_E_* = 6370 km, where *R_E_* is the Earth radius and *h* is the SAR (or aircraft) altitude [11]. As a consequence, the coordinates for a specific ellipsoid planet can be easily computed resorting to commercial software tool, such as in [14]. A pictorial view of ECEF and flat Earth (*H*) frame can be found in Figure 2b. 

**Figure 3:**
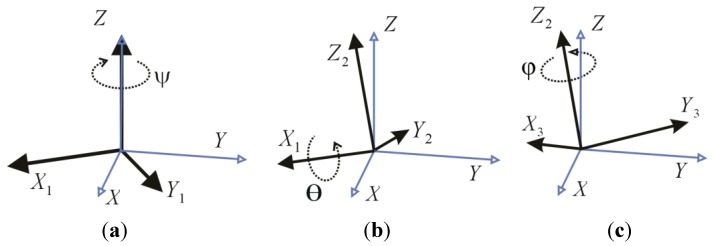
(**a**) Euler angles (*ψ*, *θ*, *φ*): (**a**) *ψ* defines the first rotation about the *z*-axis (note that the angle *ψ* in the figure is negative); (**b**) *θ* defines the second rotation about the *x_1_*-axis (note that *θ* is negative); (**c**) *φ* defines the third rotation about the *z_2_*-axis (note that *φ* is positive).

*Euler angles*: angles that are used to define the orientation of a rigid body within an inertial system of coordinates [15] (see Figure 3). Such angles hereinafter will be also referred as attitude (ψ,θ,φ). 

Now, let us define a coordinate transformation of a point $p_{H}$ from an inertial (*H*) frame to a new coordinate system (*C*) [15,16]:

(1) $$p_{C}=t_{C}+\gamma_{H}^{C}R_{H}^{C}\left(\psi ,\theta ,\varphi \right)p_{H}$$

where every quantity is defined in Table 1. This transformation has some interesting properties [15]: if the ground was planar ($z_{H}=0$), the transformation would be *affine*; $R_{H}^{C}\left(\psi ,\theta ,\phi \right)$ can be factorized into the product of three orthonormal-rotation-matrices; a parallel pair in the *H* domain remains parallel in *C*; the transformation either stretches or shrinks a vector; typical SAR distortions (*i.e.*, *foreshortening*, *layover*, *shadow* in Figure 1 and in Figure 2a) are correctly modelled [16,17]. 

Equation (1) can be inverted as follows:

(2) $$p_{H}=t_{H}+\gamma_{C}^{H}R_{C}^{H}\left(\psi ,\theta ,\varphi \right)p_{C}$$

where every quantity is defined in Table 1. The main geometrical properties considered here are: invariance to rotation of an SAR image with respect to its corresponding geographical map; invariance to scale factor (stretch or shrink); invariance to translation; invariance to slant range, foreshortening and layover distortions for θ≠0 (Figure 2b). To perform the feasibility analysis of the SAR amplitude-based geo-referencing approach, we propose a procedure relying on a well-established and simple coordinate transformation of a point from an inertial frame (*H*) to a new coordinate system (*C*), hereinafter the radar frame [16]. Equation (1) allows us to transform three-coordinates of the local flat Earth (*H*) frame into three-coordinates of the system (*C*). However, our goal is to transform a 3-coordinate system into a new two-coordinate system, where every point is a pixel of the scene imaged by the SAR. As a consequence, for the third coordinate the following condition holds:

(3) $$z_{C}=0$$

Note that, $z_{C}$ can be still computed as a function of the focal length for an optical image, but it cannot be generally derived for a SAR image if (ψ,θ,φ), $t_{C}$ and $\gamma_{H}^{C}$ are unknown. 

By exploiting Equations (1) and (2), the following relation for the aircraft position (or equivalently the SAR position) estimation can be written:

(4) $$t_{H}=p_{H}-R_{C}^{H}\left(\psi ,\theta ,\varphi \right)p_{C}$$

where it $\gamma_{C}^{H}$ was set equal to one. The previous equation clearly states that SAR position ($t_{H}=p_{H}^{\left(SAR\right)}$) can be found if and only if: SAR attitude (ψ,θ,φ) is “correctly” measured or estimated; coordinates of a point landmark ($p_{H}=p_{H}^{\left(GCP\right)}$) are known (from a DB) in the inertial coordinate system *H*; the same landmark point is imaged by the SAR and its coordinates in the non-inertial coordinate system *C*, *i.e.*, $p_{C}=p_{C}^{\left(GCP\right)}$, are correctly extracted by the ATR processing chain. 

Thus, SAR position can be computed by re-writing Equation (4) as follows:

(5) $$p_{H}^{\left(SAR\right)}=p_{H}^{\left(GCP\right)}-R_{C}^{H}\left(\psi ,\theta ,\varphi \right)p_{C}^{\left(GCP\right)}$$

An SAR sensor essentially measures the slant-range between the sensor position $p_{H}^{\left(SAR\right)}$ and a GCP, e.g.,: $p_{H}^{\left(n\right)}={\left[0,R_{n}\cos\theta_{n},0\right]}^{t}$, point A in Figure 2a located at the near range *R_n_* and corresponding to a SAR attitude (*ψ* = 0°, *θ = θ_n_*, *φ* = 0°). Thus, if the GCP is in the landmark DB (*i.e.*, $p_{H}^{\left(n\right)}$ is known) and is correctly extracted from the SAR image by the ATR chain (*i.e.*, $p_{C}^{\left(n\right)}$ is known), then the SAR sensor position $p_{H}^{\left(SAR\right)}$ can be retrieved by using Equation (5). Note also that (*ψ*, *θ_n_, φ*) have to be estimated or measured in order to exploit Equation (5). 

It is worth noting that this section does not aim to provide an SAR-amplitude based geo-referencing procedure, but only a simplified mathematical approach to perform a feasibility analysis. 

**Table 1:** Parameter classification, definition and measurement unit.

	Parameter	Definition	Measurement Unit
**Coordinate Systems**	*H*	Local flat Earth coordinate system in Figure 2b	–
	*C*	Local coordinate system, e.g., radar coordinates	–
	*h*	Altitude respect to frame *H* in Figure 2a	(km)
	$p_{H}={\left[x_{H},y_{H},z_{H}\right]}^{t}$	Position of a point in the *H* frame in Figure 2b, e.g., SAR or GCP ($p_{H}^{\left(GCP\right)}$)	(m)
	$t_{H}$	Translation vector (3 × 1) in the *H* frame	(m)
	$\gamma_{H}^{C}$	Scale parameter of the transformation from *H* to *C*	–
	$R_{H}^{C}\left(\psi ,\theta ,\phi \right)$	Rotation matrix (3 × 3) from *H* to *C* frame	–
	$p_{C}={\left[x_{C},y_{C},z_{C}\right]}^{t}$	Position of a point in the *C* frame (e.g., GCP position $p_{C}^{\left(GCP\right)}$)	(m)
	$t_{C}$	Translation vector (3 × 1) in the *C* frame	(m)
	$\gamma_{C}^{H}$	Scale parameter of the transformation from *C* to *H*	–
	$R_{C}^{H}\left(\psi ,\theta ,\phi \right)$	Rotation matrix (3 × 3) from *C* to *H* frame	–
	$p_{H}^{\left(SAR\right)},p_{H}^{\left(n\right)},p_{H}^{\left(c\right)},p_{H}^{\left(f\right)}$	*H* frame coordinates of SAR/aircraft, point A, C and B in Figure 2a	(m)
	(*ψ*, *θ*, *φ*)	Euler angles in Figure 3	(°)
**Radar**	*θ*_0_	Depression angle of radar beam-centre in Figure 2a	(°)
	*θ_inc_* *=* 90° − *θ_0_*	Incidence angle of radar beam-centre on a locally flat surface in Figure 2a	(°)
	*d*	Swath-width in Figure 2a	(km)
	Δ*θ*_0_	Beam-width in the elevation plane in Figure 2a	(°)
	Δ*_r_* , Δ*_cr_*	Image resolution along range and cross-range	(m)
	*R_n_*, *R_0_*, *R_f_*	Near, center of the beam and far range in Figure 2a	(km)
	*R_max_* or *R_f_*	Maximum detection range (or far range)	(km)
	*θ_n_*, *θ_0_*, *θ_f_*	Near, center of the beam and far depression angle in Figure 2a	(°)
**Navigation**	Δh	RALT accuracy in [%] (root mean square error—rmse)	–
	$\sigma_{\psi },\sigma_{\theta },\sigma_{\phi }$	IMU attitude accuracy on each component (rmse)	(°)
**Processing**	Δp	Inaccuracy on landmark position extraction from a SAR image	(pixel)

### 2.3. Feasibility Analysis

As already stated in Section 1, the feasibility analysis refers in particular to MALE UAV class [10], which permits heavy and wide payloads to be carried onboard, and can be affected by a dramatic cumulative drift during long mission when GPS data are not reliable. Some examples of MALE UAVs are Predator B, Global Hawk and Gray Eagle, which are actually equipped with SAR system and can be also equipped with InSAR system (more details in Section 3). Several competing system parameters have to be considered in the feasibility analysis as detailed in the following. 

X band choice is mainly driven by both the limited payload offered by X-band SAR/InSAR systems and by the wavelength robustness to “rain fading” [17]. 

Concerning the polarisation, a single channel leads to a small and light SAR system that can fit onboard a UAV. Moreover, in the case of single polarisation, well-suited statistical distribution for terrain modelling can be employed by a fast and high-performance ATR chain. Finally, backscattering in VV polarization is higher than in HH polarisation for incidence angle greater than 50° and X band [17]. 

SAR image resolution has to be chosen as a trade-off among competing requirements. Range/cross-range resolution of about 1 m is suitable for recognizing terrain landmarks, *i.e.*, large targets such as buildings, cross-roads, whose shorter linear dimension is at least 10–20 times the suggested resolution [18]. On the contrary, pixel resolution higher than 1m would be unnecessary and increase ATR computational load. 

Stripmap imaging mode allows us a shorter observation/integration time (as opposed to spotlight mode), lower computational complexity, and easier autofocusing [19]; moreover, it also requires a mechanical antenna steering mechanism simpler and lighter than other imaging modes [18]. 

Requirements on SAR acquisition geometry (*i.e.*, altitude *h* and attitude (*ψ*, *θ*, *φ*)), IMU attitude measurement, SAR swath-width, landmark characteristics, ATR accuracy, and operative scenarios, were derived through Montecarlo simulations according to the procedure described in Section 2.1.

In particular, the simulated scenarios assume good position accuracy on landmark coordinates derived by ATR chain, while exploring the various parameters in Section 2.2 to achieve the “best” aircraft position estimates through the SAR-amplitude based approach. Table 2 reports a résumé of the specific case studies presented in the following. 

The swath-width *d* is defined by the near-range *R_n_* and the far-range *R_f_* reported in Table 2 for all the explored configurations. Position estimation is based on a single GCP and computed in three cases: point A (*i.e.*, $p_{H}^{\left(n\right)}$), C (*i.e.*, $p_{H}^{\left(c\right)}$) and B (*i.e.*, $p_{H}^{\left(f\right)}$) in Figure 2a and in Table 2.

**Table 2:** Synthetic aperture radar (SAR) amplitudes-based approach: common parameter settings, case studies and results.

	SAR	Coordinates (SAR, GCPs) and Route	Source of Inaccuracy
**Common Parameters of Case Studies**	VV polarization X band Stripmap mode Δ*_r_* = Δ*_cr_* = 1 m	$p_{H}^{\left(SAR\right)}=t_{H}^{\left(SAR\right)}={\left[0,0,h\right]}^{t}$,	$\sigma_{\psi }=\sigma_{\theta }=\sigma_{\phi }\in [0.05{}^{\circ},1{}^{\circ}]$ Δ*h* = 0.5%, 1%, 2% Δ*p* = ±2, ±4
		$p_{H}^{\left(n\right)}={\left[0,R_{n}\cos\theta_{n},0\right]}^{t}$,	
		$p_{H}^{\left(c\right)}={\left[0,R_{0}\cos\theta_{0},0\right]}^{t}$,	
		$p_{H}^{\left(f\right)}={\left[0,R_{f}\cos\theta_{f},0\right]}^{t}$,	
		ψ=0°, ϕ=0°; d=2,…,10 km	
**Case Study**	**SAR/Platform Position (m)**	**Other Settings (km)**	**SAR Position Estimates**
**CS#1**	$p_{H}^{\left(SAR\right)}={\left[0,0,8000\right]}^{t}$	$R_{n}\in [45.6,\;41.7],\;R_{f}\in [46.6,\;51.5]$	Figure 4: accuracy lower than in CS#2–5, $\theta_{0}$ = 10°
**CS#2**	$p_{H}^{\left(SAR\right)}={\left[0,0,6000\right]}^{t}$	$R_{n}\in [11.6,\;9.2],\;R_{f}\in [12.5,\;18.0]$	Figure 5: $\theta_{0}$ = 30°
**CS#3**	$p_{H}^{\left(SAR\right)}={\left[0,0,6000\right]}^{t}$		Figure 6: accuracy lower than in CS#2, $\theta_{0}$ = 40°
**CS#4**	$p_{H}^{\left(SAR\right)}={\left[0,0,4000\right]}^{t}$	$R_{n}\in [7.6,\;5.7],\;R_{f}\in [8.5,\;14.7]$	Figure 7: $\theta_{0}$ = 30°
**CS#5**	$p_{H}^{\left(SAR\right)}={\left[0,0,4000\right]}^{t}$		Figure 8: accuracy lower than in CS#4, $\theta_{0}$ = 40°

We assumed that: SAR attitude (*ψ*, *θ*, *φ*) is measured by the IMU with a rmse ranging from 0.05° to 1° (without GPS correction), which is allowed by high accurate (*i.e.*, navigation grade class) commercial IMU such as LN-100G IMU [20]; *h* is measured by a RALT with accuracy equal to Δ*h* = 0.5%, 1%, 2%, which is allowed by commercial systems compliant with the regulation in [21]; SAR image resolution is Δ*_r_* = Δ*_cr_* = 1 m; inaccuracy on landmark position extraction in SAR image is Δ*p* = ±2, ±4 pixels, which is compatible with the performance of the ATR algorithms [5,8]. Finally, without loss of generality, we refer to the simplified geometry in Figure 2a defined by the following relations: $p_{H}^{\left(SAR\right)}=t_{H}^{\left(SAR\right)}={\left[x_{H}^{\left(SAR\right)},y_{H}^{\left(SAR\right)},z_{H}^{\left(SAR\right)}\right]}^{t}={\left[0,0,h\right]}^{t}$, *ψ* = 0°, *φ* = 0°. 

**Figure 4:**
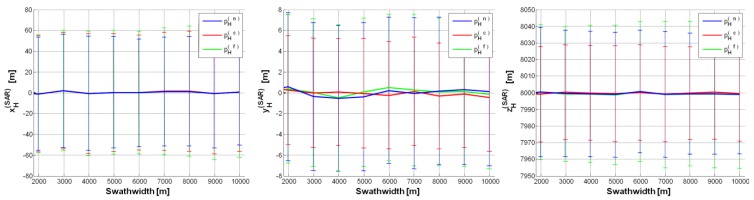
SAR position estimates (CS#1 in Table 2) based on a single GCP ($p_{H}^{\left(n\right)}$, $p_{H}^{\left(c\right)}$, $p_{H}^{\left(f\right)}$): $p_{H}^{\left(SAR\right)}={\left[0,0,8000\right]}^{t}$ m, *θ_0_* = 10°, $\sigma_{\psi }=\sigma_{\theta }=\sigma_{\phi }=0.05{}^{\circ}$, Δ*h* = 0.5%, Δ*p* = ±2. Error (on each SAR position component) mean value curve as a function of swath-width (*d*); error bars show the error standard deviation along the curve.

The first case study (CS#1 in Table 2) corresponds to a high altitude flight (*i.e.*, *h* = 8 km), which is allowed by a MALE UAV class [10]. Figure 4 depicts UAV/SAR position estimates under the “best” configuration (*i.e.*, *θ_0_* = 10°, $\sigma_{\psi }=\sigma_{\theta }=\sigma_{\phi }=0.05{}^{\circ}$, Δ*h* = 0.5%, Δ*p* = ±2), with error bars proportional to the error standard deviation (std): no significant bias can be noted, but the std, which is very stable w.r.t. the swath-width, is too large on the 1st and 3rd component ($x_{H}^{\left(SAR\right)},y_{H}^{\left(SAR\right)}$). Even by increasing *θ_0_* (from 10° to 40°), suitable results cannot be achieved. Note that if the IMU rmse was $\sigma_{\psi }=\sigma_{\theta }=\sigma_{\phi }=0.5{}^{\circ}$, the std of each component of $p_{H}^{\left(SAR\right)}$ would be about 10 times greater than in Figure 4 (where IMU rmse = 0.05°). Analogously, if RALT accuracy was Δ*h* = 1% and 2%, the std of both the 2nd and 3rd component of the estimated SAR position would be about 1.25 and 2.5 times greater than in Figure 4 (where Δ*h* = 0.5%). On the contrary, inaccuracy on landmark position in the image (derived by the ATR chain) has no appreciable impact on SAR position estimates (e.g., Δ*p* = ±2, ±4). 

**Figure 5:**
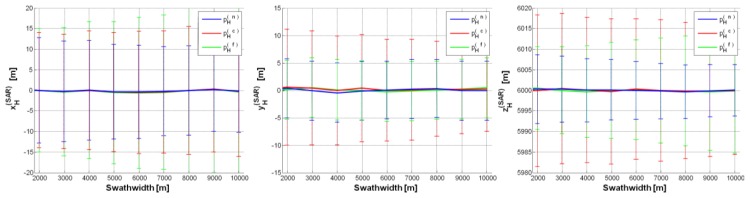
SAR position estimates (CS#2 in Table 2) based on a single GCP ($p_{H}^{\left(n\right)}$, $p_{H}^{\left(c\right)}$, $p_{H}^{\left(f\right)}$): $p_{H}^{\left(SAR\right)}={\left[0,0,6000\right]}^{t}$ m, *θ*_0_ = 30°, $\sigma_{\psi }=\sigma_{\theta }=\sigma_{\phi }=0.05{}^{\circ}$, Δ*h* = 0.5%, Δ*p* = ±2.

Thus, to reach suitable estimates, we decreased the SAR altitude to *h* = 6000 m (case study CS#2 in Table 2). The results from the “best” configuration are shown in Figure 5. It can be seen that all the estimated components of $p_{H}^{\left(SAR\right)}$ have no bias and their variability is always bounded within ±20 m. Fairly worse results are achieved by exploiting $p_{H}^{\left(c\right)}$ as landmark, because of the higher uncertainty on the corresponding depression angle *θ_c_*. Moreover, the estimates of $p_{H}^{\left(SAR\right)}$ based on $p_{H}^{\left(n\right)}$ are generally better than those based on $p_{H}^{\left(f\right)}$, because the impact of IMU inaccuracy is stronger on *θ_f_* than on *θ_n_* (*θ_f_* < *θ_n_*). 

**Figure 6:**
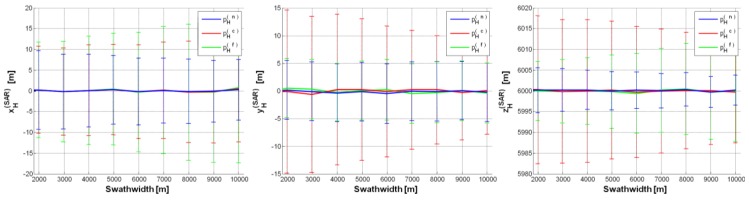
SAR position estimates (CS#3 in Table 2) based on a single GCP ($p_{H}^{\left(n\right)}$, $p_{H}^{\left(c\right)}$, $p_{H}^{\left(f\right)}$): $p_{H}^{\left(SAR\right)}={\left[0,0,6000\right]}^{t}$ m, *θ_0_* = 40°, $\sigma_{\psi }=\sigma_{\theta }=\sigma_{\phi }=0.05{}^{\circ}$, Δ*h* = 0.5%, Δ*p* = ±2.

**Figure 7:**
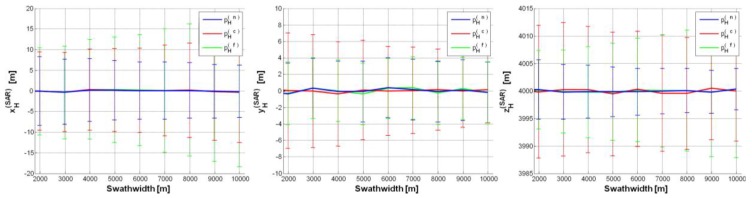
SAR position estimates (CS#4 in Table 2) based on a single GCP ($p_{H}^{\left(n\right)}$, $p_{H}^{\left(c\right)}$, $p_{H}^{\left(f\right)}$): $p_{H}^{\left(SAR\right)}={\left[0,0,4000\right]}^{t}$ m, *θ_0_* = 30°, $\sigma_{\psi }=\sigma_{\theta }=\sigma_{\phi }=0.05{}^{\circ}$, Δ*h* = 0.5%, Δ*p* = ±2.

**Figure 8:**
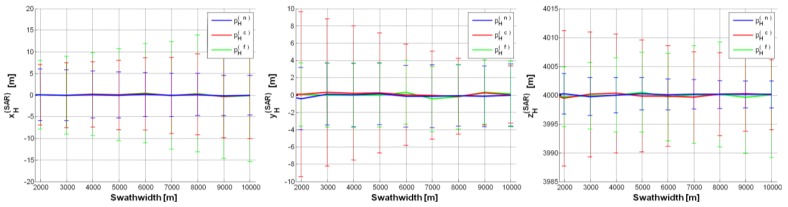
SAR position estimates (CS#5 in Table 2) based on a single GCP ($p_{H}^{\left(n\right)}$, $p_{H}^{\left(c\right)}$, $p_{H}^{\left(f\right)}$): $p_{H}^{\left(SAR\right)}={\left[0,0,4000\right]}^{t}$ m, *θ_0_* = 40°, $\sigma_{\psi }=\sigma_{\theta }=\sigma_{\phi }=0.05{}^{\circ}$, Δ*h* = 0.5%, Δ*p* = ±2.

We also considered a case study (CS#3 in Table 2) with greater SAR depression angle (*θ_0_* = 40°), while keeping platform *h* as in CS#2. Figure 6 show CS#3 results, which keep on satisfying the constraint on position accuracy. Note that the accuracy on $x_{H}^{\left(SAR\right)}$ is improved compared with CS#2, but it is worse on $y_{H}^{\left(SAR\right)}$, because of the wider Δ*θ_0_* for *θ_0_* = 40° than for *θ_0_* = 30° (*i.e.*, CS#2) needed to illuminate the same swath-width. For depression angles higher than 40°, the accuracy on the estimates further worsens: such a trend can be also observed by comparing Figure 5 and Figure 6. 

As a limiting case, we reduced the UAV altitude down to *h* = 4000 m (CS#4 in Table 2). Figure 7 shows position estimates under the “best” configuration (*θ_0_* = 30°): the estimated $p_{H}^{\left(SAR\right)}$ components show negligible bias and their variability is even lower than in the previous case studies, *i.e.*, ±15 m. Note that, for swath-width greater than 7000 m, only $x_{H}^{\left(SAR\right)}$ exceeds the previous boundary. 

We also considered a further case study (CS#5 in Table 2) with a depression angle *θ_0_* = 40° greater than in CS#4. All the estimates shown in Figure 8 are rigorously bounded within ±15 m and SAR position estimates are generally very similar to those achieved in CS#4. Only the inaccuracy on $y_{H}^{\left(SAR\right)}$ slightly increases, because of the wider Δ*θ_0_* for *θ_0_* = 40° than for *θ_0_* = 30°. Again, for depression angles higher than 40°, the accuracy on the estimates further worsens.

A UAV altitude lower than 4000 m cannot be taken into account because it would have severe consequences on both SAR system requirements and SAR data processing. In fact, in order to keep constant the values of both *θ_0_* and swath-width, the lower the altitude, the wider Δ*θ_0_*, thus leading to considerably increasing the required transmitted power. Moreover, a wide Δ*θ_0_* means large resolution changes across the ground swath [8], which negatively impact the performance of the ATR algorithm: features that are clearly distinguishable at far range can become nearly invisible at near range. 

In conclusion, feasible parameter settings are those relative to CS#2 and CS#4 configurations, which provide estimated UAV coordinates with errors bounded within ±18 m and ±12 m, respectively. In Table 3, a résumé of the suggested requirements, *i.e.*, a first trade-off, is listed. Note that the suggested range of SAR swath-width *d* (*i.e.*, few kilometres) allows us to be confident about the presence of the desired landmarks within the illuminated area, even if the SAR system (due to uncertainty on attitude and position) points at the wrong direction. It is worth noting that SAR requirements in Table 3 can be easily fulfilled by commercial systems, e.g., Pico-SAR radar produced by Selex-ES [22]; MALE UAV [10] such as Predator B, Global Hawk and Gray Eagle, which easily carry a Pico-SAR radar; navigation grade class IMU such as LN-100G IMU [20]; any RALT compliant with the regulation in [21].

**Table 3:** Platform, viewing geometry, navigation sensor requirements and corresponding reference system which allows a feasible SAR position retrieval: *first trade-off*.

	First Setting (CS#2)	Second Setting (CS#4)
Aircraft altitude (km)	6	4
Depression angle *θ_0_* (°)	30	30
Swath-width *d* (km)	few units	few units
Elevation beam-width Δ*θ_0_* (°)	≥11.5	≥16.6
Image resolution (m)	~1	
SAR band	X	
SAR polarization	VV	
*R_n_* (km); *R_0_* (km); *R_f_* (km)	**10.3**−9.2; 12; **14.6**−18.0	**6.5**−5.7; 8; **10.8**−14.7
Maximum detection range *R_f_* (km)	≥14.6	≥10.8
Δ*h* (%)	0.5 (or fairly worse)	0.5 (or fairly worse)
$\sigma_{\psi },\sigma_{\theta },\sigma_{\phi }$ (°)	0.05	0.05
Estimated UAV based on $p_{H}^{\left(c\right)}$ (bias ± std) (m)	0 ± 15, 0 ± 11, 0 ± 18 (Figure 5)	0 ± 12.5, 0 ± 7.5, 0 ± 12 (Figure 7)
**Reference systems**	MALE UAV, e.g.,: Predator B, Global Hawk, Gray Eagle	
	Pico-SAR	
	Any RALT compliant with the regulation in [21]	
	Navigation grade class IMU, e.g.,: LN-100G IMU	

### 2.4. Landmark DB and Mission Planning

In order to retrieve SAR position, the proposed algorithm has to exploit landmark points. In the previous section, it is assumed that landmark coordinates in the mission DB are ideal, *i.e.*, without errors. Such an assumption is quite unlikely, and, consequently, we also evaluated the impact of landmark coordinate inaccuracy on the estimated UAV position. According to the previous analysis, preliminary requirements were derived for the landmark DB reference domain, typology, and accuracy. Concerning the reference domain, an inertial system of coordinates (*H*) can be adopted, e.g., Earth-centered frame or local map coordinate system. Concerning the landmark typology, planar landmarks (e.g., crossroad, roundabout, railway crossing) are strongly suggested because they are very recurrent in several operating scenarios, can be precisely extracted, and do not introduce any vertical distortion due to elevation with respect to the ground surface [8]. Small 3D-landmarks can be also exploited but in the limits of the visibility problems due to the shadow areas occurring close to high structures. According to this, a mission planning should avoid scenarios densely populated by buildings, while preferring suburban or rural areas.

Concerning landmark DB accuracy, we exploited the procedure in Section 2.2 and the most promising two settings in Section 2.3. Table 4 presents the platform geo-referencing accuracy derived under two different error settings: a “moderate” DB accuracy with RMSE equal to [3, 3, 3]*^t^* in (m) on landmark coordinates of $p_{H}^{\left(c\right)}$, and a “low” DB accuracy with RMSE equal to [3, 3, 10]*^t^* in (m). Results show a limited increase in the final std values with respect to the ideal results in Section 2.2. 

The DB accuracy assumed above is quite reasonable, because the coordinates of interest refer to centroid and corners of small landmarks, and can be derived by exploiting a Geographic Information System (GIS) archive (or similar) [23]. The mapping standards employed by the United States Geological Survey (USGS) specifies that [24]: 90% of all measurable horizontal (or vertical) points must be within ±1.01 m at a scale of 1:1200, within ±2.02 m at a scale of 1:2400, and so on. Finally, the errors related to the geo-referencing could be modelled as a rigid translation of a landmark with respect to its ideal position, with RMSE smaller than 10 m. 

**Table 4:** Platform position retrieval (bias ± standard deviation of each coordinate) as a function of the settings in Table 1 and Data Base (DB) accuracy on landmark coordinates of $p_{H}^{\left(c\right)}$.

Landmark DB Accuracy	CS#2	CS#4
“Moderate“ (rmse [3, 3, 3]*^t^* in (m))	0 ± 14.8 m, 0 ± 10.2 m, 0 ± 17.8 m	0 ± 12.4 m, 0 ± 05.8 m, 0 ± 12.1 m
“Low“ (rmse [3, 3, 10]*^t^* in (m))	0 ± 14.6 m, 0 ± 10.2 m, 0 ± 20.2 m	0 ± 12.4 m, 0 ± 05.8 m, 0 ± 15.3 m

## 3. InSAR Phase Exploitation

**Figure 9:**
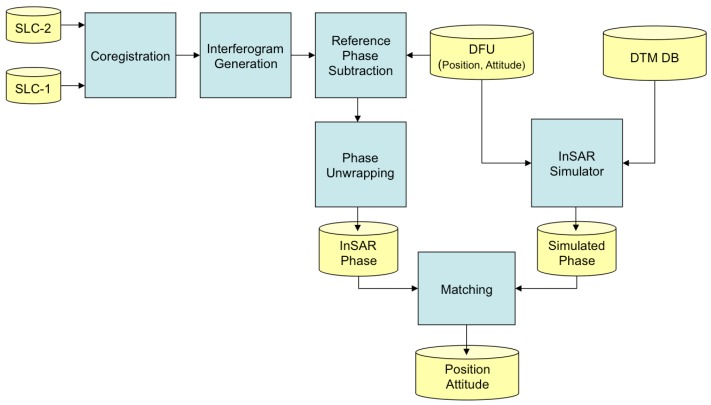
Flow chart of the InSAR based navigation system.

When the use of SAR amplitude is unfeasible because of unreliable surveyed scenario, the InSAR phase can be exploited as derived by a single pass interferometer mounted onboard the platform. The proposed approach is sketched in Figure 9 and basically consists in comparing the real InSAR phase with a synthetic one derived by using both approximated position and attitude values and a reference DTM. The matching block provides a feedback to be used for adjusting position and attitude values derived by the instruments available onboard. 

### 3.1. InSAR Setting 

**Figure 10:**
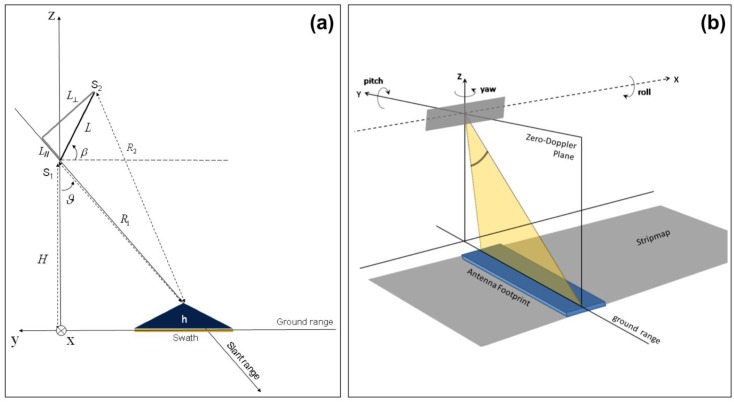
(**a**) InSAR acquisition geometry; (**b**) UAV position (X, Y, Z) and attitude (roll, pitch, yaw) in the basic SAR geometry.

According to the acquisition geometry sketched in Figure 10, the phase difference (Φ) between two slightly displaced SAR observations S1 and S2 is related to the difference in time delay from a given pixel to each antenna of the SAR Interferometer. In the hypothesis of bistatic acquisitions (real interferometer), the InSAR phase is not affected by neither atmosphere, nor ground deformation. It depends only on the geometrical distance and can be expressed in terms of the reference phase (Φ_ref_), which accounts for the difference between the slant range geometry and the reference elevation model (ellipsoidal or flat), while the topographic phase (Φ_h_) is related to the elevation *h* w.r.t. the reference elevation model [25]:

(6) $$\Phi (p)=\Phi^{ref}(p)+\Phi^{h}(p)\approx \frac{2\pi }{\lambda }\cdot \left[L_{//,p}-\frac{h(p)}{h_{a}}\right]$$

where *λ* is the SAR wavelength, *L*_//,*p*_ is the component parallel to the master slant direction of the geometrical distance between the two acquisitions (or baseline, *L*), and *h_a_* is the so-called *height of ambiguity.* This last is defined as:

(7) $$h_{a}=\frac{\lambda R_{S1,p}sen\left(\theta_{p}-\alpha_{p}\right)}{L_{⊥,p}}$$

where *R_S1,p_* and *θ_p_^ref^* are, respectively, the range distance and the look angle relative to the master (S1) view computed w.r.t. the reference elevation at the pixel *p*; *L*_⊥,*p*_ is the component of the baseline orthogonal to the slant direction of the master computed at pixel *p*; *α_p_* is the terrain slope. *h_a_* defines the InSAR height sensitivity, depends on the SAR system specifications, and drives the performance of the InSAR processing for height computation. In particular, the smaller the value of the height of ambiguity, the smaller the detectable height difference.

Since *h_a_* is inversely proportional to the effective baseline *L*_⊥_, high baseline values are recommended for accurate topography mapping. Basically, the aircraft size and shape limit the baseline values and consequently *h_a_*. The baseline component *L*_⊥_ depends also on both the look angle and the orientation angle *β* (see Figure 10), which can be optimized in order to maximize *L*_⊥_. In the following, we assume a vertical baseline (*β =* 90°), which guarantees a symmetric weight distribution across the aircraft fuselage. An inclination angle *β* equal to the mean look angle (*β = θ* in Figure 10) maximizes the orthogonal component of the baseline *L*_⊥_ leading to increase the height sensitivity up to 5% for low incident angle.

The following analysis was carried out assuming, as in the case of amplitude-based procedure, a MALE UAV class [10], which can carry on a maximum payload of 150 × 130 cm^2^ and reach a maximum flight altitude of 8000 m.

### 3.2. Analysis with Respect to InSAR Phase Integrity

The reliability of the InSAR processing depends on the quality of the InSAR phase, which is related to the correlation between the two acquisitions. Changes of the terrain conditions between the two acquisitions, system noise, and approximations in the processing lead to correlation loss. The image correlation can be assessed in terms of the InSAR coherence *γ* [9], which is a normalized correlation coefficient varying from 0 (full decorrelation) to 1 (full correlation). The coherence is directly related to the signal to noise ratio of the interferogram and can be partitioned according to different sources of decorrelation. In particular, the geometric decorrelation, *δ_geo_*, is due to the difference between the incidence angles of the two acquisitions. This geometrical difference causes a spectral shift between the signal spectral bands, which is proportional to the effective geometrical baseline [26]. When the spectral shift equals the signal bandwidth the SAR images are totally uncorrelated and the baseline has a value known as critical baseline. 

The volume decorrelation, *δ_vol_*, is due to the penetration of the radar waves into the soil or vegetation and depends on both radar wavelength and scattering medium (bare soil, urban structures, vegetation or forest) [27]. The volume scattering can be neglected in case of low penetration (bare soil) or high extinction rate (forested area) as for X band or higher. In the following we assume to refer to an X band system thus allowing us to neglect the volume decorrelation. This choice is also able to guarantee better height sensitivity, as from Equation (7). 

A further important source of errors comes from the fact that the InSAR phase field is obtained by extracting the phase term of the complex interferogram leading to an ambiguity of 2π in the real phase measurement. The Phase Unwrapping (PU) consists in deriving the correct map of the multiples of 2π to be added to the “wrapped” phase in order to infer the absolute phase field correlated to the ground topography images [28]. This processing step is critical in terms of both computational needs and reliability. SAR systems at high spatial resolution, smooth terrains, and proper baselines can help in performing a reliable PU. In particular, the height of ambiguity, which depends on the baseline value according to Equation (7), provides also the sensitivity to the wrapping of the InSAR phase. Thus, large baseline values improve the accuracy on height estimations, but also lead to a wide range of terrain slopes affected by aliasing. According to the geometric and radiometric system parameters, the critical slope angle *α_C_* can be defined so that if the terrain slope *α* is in the interval [*θ − α_C_*, *θ + α_C_*], then the InSAR phase is affected by aliasing and PU fails. 

Therefore, height of ambiguity, geometrical decorrelation and critical angle are useful indicators of the expected performances of the interferometer. The first one provides the InSAR sensitivity to the height variation. The second impacts on the InSAR phase noise (usually estimated through the coherence) which causes artifacts in the phase unwrapping, thus decreasing the accuracy of the final measurement. The latter provides a direct indication on the areas where phase unwrapping could fail. 

We explored these three figures to derive reliable radiometric and geometric parameters for the InSAR configuration. In particular, high baselines are required for increasing the InSAR sensitivity to the height; however, at the same time, they increase the geometrical decorrelation as well as the probability of phase aliasing to occur, thus making PU more problematic. High carrier frequencies, or equivalently short wavelengths, increase the geometrical decorrelation but in general ensure less penetration, thus limiting the effect of volumetric decorrelation.

Moreover, by increasing the bandwidth, or equivalently the spatial resolution, the geometrical decorrelation (and consequently the critical angle *α_C_*) decreases as well, thus leading to improved performances of the interferometer. 

**Figure 11:**
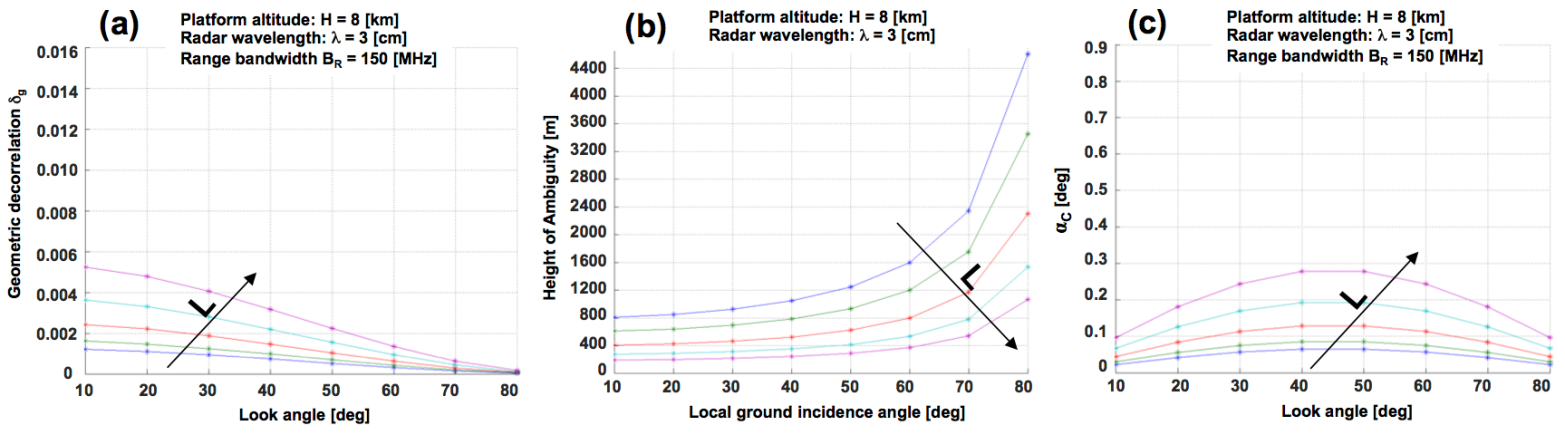
(**a**) Height of ambiguity *h_a_*, (**b**) geometric decorrelation *δ_geo_*, and (**c**) critical angle *α_C_ vs.* look angle for *B* = 150 MHz, *λ* = 3 cm , platform altitude *H* = 8 km, vertical baseline *L* = [0.3, 0.4, 0.6, 0.9, 1.3] m (from blue to purple), baseline orientation angle *β* = 90° (vertical orientation see Figure 2).

Figure 11 shows the behavior of *δ_geo_*, *h_a_* and *α_C_* with respect to different values of vertical baseline and incident angle. We simulated a bistatic InSAR system with: baseline *L* = [0.3, 0.4, 0.6, 0.9, 1.3] m, orientation angle *β* = 90° (*i.e.*, vertical orientation—see Figure 10a), aircraft elevation of 8 km, look angles ranging from 10° up to 80°. Moreover, we assumed a SAR sensor with high spatial resolution, B = 150 MHz, working at X band. Figure 11a shows the trends for *δ_geo_*. In this case, the performance improves (*δ_geo_* decreases) by increasing the look angle and by decreasing the baseline *L*. In general, the geometrical decorrelation is very limited (<0.006) for every configuration. In case of bistatic acquisitions (no temporal decorrelation) and short wavelengths (no volume decorrelation), the InSAR coherence is expected to be quite high except for the noise related to both processing (autofocusing and co-registration) and electronic devices. Figure 11b shows the trends for *h_a_*. For a fixed incidence angle, the InSAR height sensitivity increases (or equivalently *h_a_* decreases) as the baseline *L* increases. Moreover, the sensitivity decreases by increasing the look angle. In general, the sensitivity can be quite limited and, for some configurations, *h_a_* is really too high (>1000 m) to guarantee reliable performances (<1/100 rad). Figure 11c shows the trends for *α_C_*. In this case, the performance improves (*α_C_* decreases) by decreasing the baseline *L* and it is not monotonic with the look angle. However, the values of *α_C_* are always less than 1°, leading to very limited range of terrain slopes forbidden due to phase aliasing. Thus, the major problem in conventional SAR interferometry (*i.e.*, the reconstruction of absolute phase values starting from the InSAR wrapped principal phase fields, alias PU) is strongly reduced. 

From this preliminary investigation we can conclude that the explored configurations are not problematic in terms of both geometrical decorrelation and phase unwrapping. Thus, the height sensitivity should drive the selection of requirements for both radiometric and geometric parameters. In particular, high baseline values should be preferred. In the present case, the baseline value is limited by the aircraft size and shape (also considering possible external aerodynamics appendices).

### 3.3. Analysis with Respect to Altitude and Position Changes 

In the previous section, we assessed the feasibility of the proposed approach (sketched in Figure 9) with respect to the quality of the InSAR phase field. The same assessment is required with respect to the InSAR sensitivity to changes on aircraft position and attitude, which are the parameters to be estimated. To this aim, we refer to the SAR looking geometry sketched in Figure 10b (body frame, or b-frame), where: the azimuth direction defines the X coordinate, the Y axis is opposite to the ground range direction (assuming right looking systems), the Z axis is coincident with the local zenith direction, the roll error angle is measured in the YZ plane from the Z axis towards the Y axis, the yaw error angle is measured counter-clockwise in the XY plane from the X axis, the pitch error angle is defined in the XZ plane from the nadir direction towards the X axis.

We developed a geometrical model that, referring to a point target on the ground with topographic height *h*, relates changes on aircraft attitude/position to changes on both InSAR phase and target SAR coordinates (slant range, azimuth). According to this model, we investigated the effects that errors in the UAV attitude/position have on both the InSAR phase and the slant range/azimuth misalignments. The same parameters of the previous analysis were assumed (*H* = 8 km; *λ* = 3 cm). Incidence angle values between 15° and 60° were explored in order to limit the height of ambiguity. As we will see, the influence of the topographic height on the sensitivity analysis is very poor and it is almost negligible for high incidence angles (*θ* ≈ 60°).

**Figure 12:**
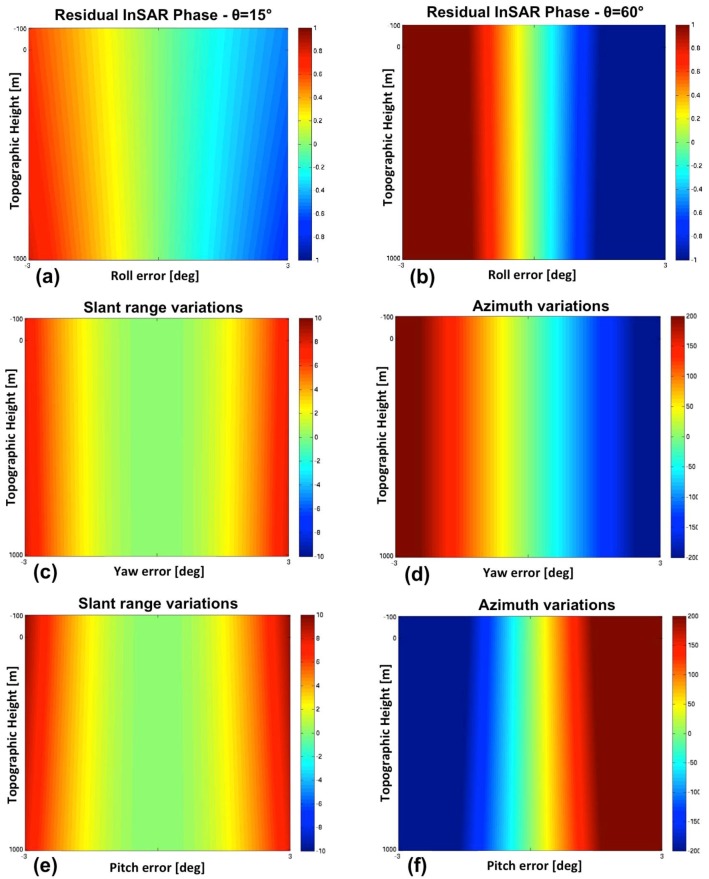
Subplots (**a**,**b**) show residual InSAR phase cycles evaluated for roll errors between −3° and 3°, and incident angles at near range (**a**) and far range (**b**). Subplots (**c**–**f**) show the misalignments in slant range and azimuth evaluated for yaw (**c**,**d**) and pitch errors (**e**,**f**) between −3° and 3°, assuming topographic heights ranging from −100 m to 1 km, and an incident angle at mid-range (*θ_inc_* = 30°). For all the plots, the topographic height ranges from −100 m to 1 km.

The sensitivity of the InSAR phase to roll errors does not vary appreciably with the platform height, while it increases with the incidence angle, and, as expected, is heavily affected by the baseline length. Plots (a) and (b) in Figure 12 show, respectively for incident angles at near (*θ* = 15°) and far range (*θ* = 60°), the residual InSAR phase cycles evaluated for roll errors between −3° to 3°, by assuming baseline *L* = 1.3 m and topographic heights ranging from −100 m to 1 km. With regard to the sensitivity on the position of the selected target, the analysis is restricted to the slant range, since a roll error cannot introduce a misalignment along the azimuth direction. Roll errors even up to 3° (in absolute value) lead to slant range variations less than 1 m. Hence, the slant range sensitivity is very poor in this case. 

Concerning errors on the yaw angle, the analytical model predicts a poor sensitivity of the InSAR phase and a high sensitivity of the azimuth misalignment, which increases with both incident angle and aircraft altitude. In particular, a yaw error of just 1° may lead to azimuth shifts close to 200 m (see Figure 12d), with incidence angles close to 60°. The slant range sensitivity is almost independent from the aircraft altitude and it slightly increases with the incidence angle, while a yaw error of 1° never produces a slant range variation exceeding 2 m (see Figure 12c) for all the explored configurations. Moreover, an intrinsic uncertainty on the sign of the yaw error affects the estimation based on slant range misalignment. 

As for the yaw angle, sensitivity of InSAR phase to errors on pitch angle is generally very poor. Also the slant range misalignment is very low in particular for pitch errors not exceeding 1° (see Figure 12e). On the contrary, azimuth misalignments are important, regardless the platform height and the incidence angle. 1° pitch error may lead to azimuth misalignments higher than 200 m, as shown in Figure 12f. Errors on the along-track coordinate (*i.e.*, X coordinate) do not affect the InSAR phase or the Zero-Doppler distance between the target and the sensor after SAR focusing. They only lead to a corresponding azimuth misalignment in the focused image.

**Figure 13:**
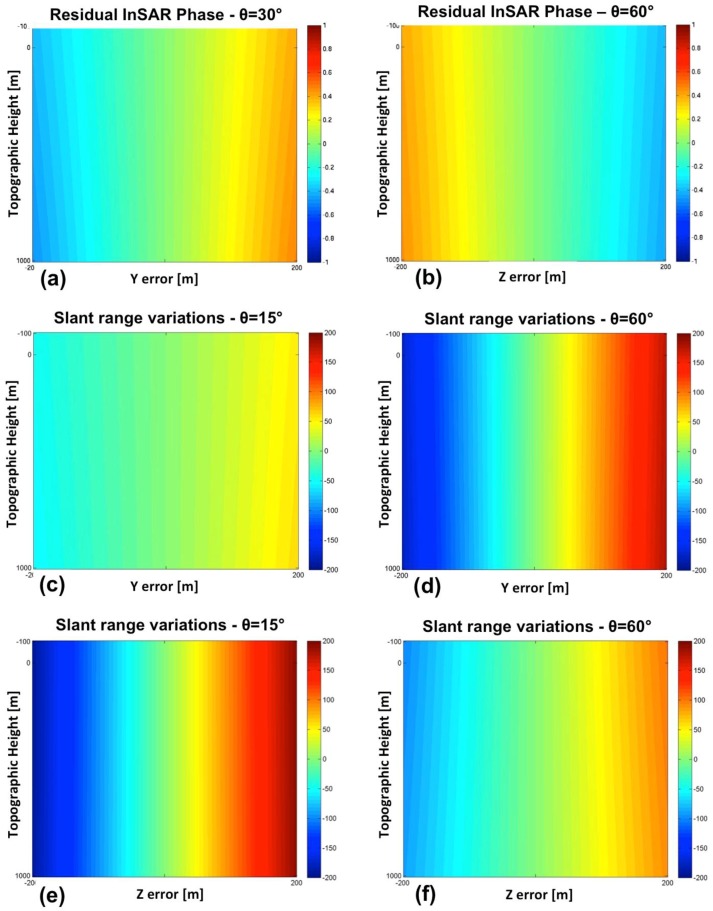
Subplots (**a**) and (**b**) show residual InSAR phase cycles evaluated for Y errors (**a**) and Z errors (**b**) ranging from −200 to 200 m, and incident angles at mid-range and far range respectively. Subplots (**c**–**f**) show the variation of the target position along slant rage (meters) evaluated for Y errors (**c**,**d)** and Z errors (**e**,**f**) ranging from −200 to 200 m, and for incident angle at near range (**c**,**e**) and far range (**d**,**f**). For all the plots, the topographic height ranges from −100 m to 1km.

Let us consider now an error only on the UAV position, *i.e.*, on the Y coordinate (ground range in Figure 10a). The residual InSAR phase can be computed analytically through geometrical modeling. The sensitivity of the InSAR phase is high for low orbits and at mid-range, and it increases significantly with the baseline length (Equation (6)). In general for a platform altitude of 8 km the InSAR phase sensitivity is quite limited (see Figure 13a). Concerning sensitivity of the target position to Y errors, the analysis is restricted to the slant range, since an error on the Y position cannot introduce a misalignment along the azimuth direction. Slant range sensitivity significantly increases with the incidence angle (see plots (c) and (d) in Figure 13 derived at near and fare range, respectively), while the effect of the platform altitude is negligible. Errors in slant range up to 200 m can occur. 

The last parameter to investigate is the Z position of the UAV platform. In this case, the sensitivity of the InSAR phase is high for low orbits and at far range (see Figure 13b), and again it increases significantly with the baseline length. In this case, the analysis of the sensitivity on the position of a selected target to Z errors is also restricted to the slant range, since an error on the Z position cannot introduce a misalignment along the azimuth direction. Slant range sensitivity is in general considerable and increases at near range (see plots (e) and (f) in Figure 13), while the effect of the platform altitude is negligible, as for the Y parameter.

### 3.4. Analysis with Respect to DTM Characteristics

The performances of the InSAR-based geo-referencing procedure depend on the Matching algorithm, which, in turn, depends on the InSAR height sensitivity, the spatial variability of the terrain profile and the specifications of the reference DTM available onboard. The implementation of a specific matching algorithm is beyond the scope of this paper, but a possible processing scheme can designed, based on SIFT technique [29] and, in general, strong spatial variability is required in order to improve the correlation performance. Thus, it is expected that the InSAR-based geo-referencing procedure would be reliable on hilly or mountainous areas, and when using the configuration with the highest baseline value (1.3 m in our simulated scenario), which ensures the best InSAR sensitivity to the terrain elevation.

Both spacing of the grid points and accuracy of the elevation values determine the quality of a DTM. For our purposes, a reliable DTM spacing should be comparable to the spatial resolution of the InSAR products. This, in general, is worse than the resolution of the original SAR images because of the multi-viewing required to decrease the phase noise. The number of looks adopted in this smoothing procedure can be set to the minimum value, which provides a multi-looked resolution cell comparable to the horizontal geolocation accuracy. Assuming the most favorable InSAR configuration of L = 1.3 m, and an InSAR coherence of 0.9, a reliable value for the number of looks is about four, both in range and azimuth. 

The impact of the DTM spacing on the InSAR-based geo-referenced procedure was evaluated by using the difference between the simulated InSAR phase fields derived by using DTM with different postings. A statistical analysis was performed by computing the std of the InSAR phase errors. Assuming the most favorable configuration of L = 1.3 m, and a multi-look value of 4 × 4, the DTM spacing does not exceed 10 m. This result meets the specifications of the Level 3 DTM standard defined by the National Imagery and Mapping Agency (NIMA) [30] as: spatial resolution of 12 m, absolute vertical accuracy less than 10m, relative vertical accuracy less than 2 m.

The requirement on the DTM height accuracy can be derived by considering as reliable a value at least comparable to the InSAR height sensitivity. Assuming the most favorable configuration of L = 1.3 m, and an InSAR coherence of 0.9, the height sensitivity is in general lower than 10 ÷ 13 m, depending on the look angle. Therefore, a DTM of Level 3 for the NIMA standard again meets the requirements for the height accuracy. 

Finally, the TanDEM-X mission is generating a DEM fulfilling these requirements [31] and covering the whole Earth surface, so that reliable input data to the InSAR-based geo-referencing procedure will be soon available globally.

## 4. Conclusions

The goal of the paper is to propose an advanced integration of SAR imaging into UAV navigation systems and to prove the technological feasibility of such integrated system. We address the problem of drifts in planned trajectory due to inaccurate INS measurements occurring when a GPS signal is absent or corrupted by either intentional or unintentional interferences. This problem can affect in particular the MALE UAV class during a long endurance flight. However, the same UAV class permits to carry out onboard heavy and wide payloads, thus making feasible the use of SAR/InSAR technology. SAR sensors are valuable for a UAV navigation backup system thanks to the day/night and all-weather imaging, and to the wide area illuminated from a long distance by ensuring high spatial resolution.

A feasibility analysis was aimed at deriving requirements for the geo-referencing procedure performed on a UAV platform and based on the SAR amplitude (if terrain landmarks are available) and on the SAR Interferometry (if terrain landmarks are not available). 

Simple and well-established approaches were presented to derive a feasibility analysis concerning both the amplitude-based and the InSAR-based procedures, and considering different competitors: SAR system, image acquisition geometry, navigation instruments, UAV platform, landmarks and DTMs. 

Results detailed in Section 2.4, 2.5 and 3 finally show that both SAR amplitude- and InSAR-based approaches here proposed are technologically feasible by resorting to a certain class of UAV platform, IMU, RALT, both SAR and InSAR systems, typical landmark position accuracy and class (e.g., buildings and road network in a suburban/rural scenario), and DTM accuracy. The requirements derived from the feasibility analysis can be fulfilled by commercial systems e.g., Pico-SAR radar produced by Selex-ES [22]; MALE UAV [10] such as Predator B, Global Hawk and Gray Eagle, which easily carry a Pico-SAR radar; navigation grade class IMU such as LN-100G IMU [20], and any RALT compliant with the regulation in [21]. Furthermore, the size of these UAVs permits hosting SAR interferometric system with baseline values able to provide reliable performances in terms of both phase noise and sensitivity. We also proved that a DTM of Level 3 for the NIMA standard meets the requirements for both DTM spacing and height accuracy. Moreover, a global DTM of such quality will be soon available thanks to the TanDEM-X mission. 

Concerning the amplitude-based approach, feasible parameter settings are those relative to CS#2 and CS#4 configurations in Table 3, which provide estimated aircraft coordinates with errors bounded within about ±15 m. It can be clearly stated that the parameter settings or requirements (here derived) are more focused on aerial platform, SAR systems and navigation sensors than on the image processing chain (SAR data Autofocusing, ATR chain), which is out of the scope of this paper but already exploited also for UAVs (e.g., [5,6]). 

Concerning the InSAR-based approach, the analysis showed that the phase variation does not seem to be a useful figure to correct position and attitude. On the contrary, the exploration of SAR coordinates variation due to changes in position and attitude of the aircraft appears feasible. This result suggests that, for the matching algorithm, it seems promising to explore the difference in range and azimuth location between the real and simulated InSAR phase profiles, instead of looking at the phase differences. A possible approach can be based on SIFT technique [29].

Future work will be devoted to develop an *ad hoc* and performing ATR chain (for the amplitude-based approach) and a matching algorithm (for the InSAR-based approach), as well as to process real data.

## References

[1] Chiang K., Tsai M., Chu C. (2012). The Development of an UAV Borne Direct Georeferenced Photogrammetric Platform for Ground Control Point Free Applications. Sensors.

[2] McLean D. (1990). Automatic Flight Control Systems.

[3] Hasan A.M., Samsudin K., Ramli A.R., Azmir R.S., Ismaeel S.A. (2009). A Review of Navigation Systems (Integration and Algorithms). Aust. J. Basic Appl. Sci..

[4] Greco M., Querry S., Pinelli G., Kulpa K., Samczynski P., Gromek D., Gromek A., Malanowski M., Querry B., Bonsignore A. SAR-based Augmented Integrity Navigation Architecture: SARINA project results presentation. Proceedings of 13th IEEE International Radar Symposium (IRS).

[5] González-Partida J., Almorox-González P., Burgos-Garcia M., Dorta-Naranjo B. (2008). SAR System for UAV Operation with Motion Error Compensation beyond the Resolution Cell. Sensors.

[6] Aguasca A., Acevo-Herrera R., Broquetas A., Mallorqui J., Fabregas X. (2013). ARBRES: Light-Weight CW/FM SAR Sensors for Small UAVs. Sensors.

[7] Curlander J.C., McDonough R.N. (1991). Synthetic Aperture Radar: Systems and Signal Processing.

[8] Oliver C., Quegan S. (2004). Understanding Synthetic Aperture Radar Images.

[9] Rosen P.A., Hensley S., Joughin I., Li F.K., Madsen S.N., Rodriguez E., Goldstein R.M. (2000). Synthetic Aperture Radar Interferometry. IEEE Proc..

[10] Weibel R.E., Hansman R.J. Safety Considerations for Operation of Different Classes of UAVs in the NAS. Proceedings of the AIAA 3rd “Unmanned Unlimited” Technical Conference, Workshop and Exhibit.

[11] Barton D.K. (2013). Radar Equations for Clutter and Jamming. Radar Equations for Modern Radar.

[12] (2000). World Geodetic System 1984: Its Definition and Relationships with Local Geodetic Systems.

[13] Etkin B., Reid L.D. (1972). The Stability Derivatives. Dynamics of Atmospheric Flight, Stability and Control.

[14] Matlab. http://it.mathworks.com/help/aerotbx/ug/lla2flat.html.

[15] Siciliano B., Sciavicco L., Villani L., Oriolo G. (2009). Kinematics. Robotics—Modelling, Planning and Control.

[16] Glasbey C.A., Mardia K.V. (2001). A penalized approach to image warping. J. R. Stat. Soc. Ser. B Stat. Methodol..

[17] Richards J.A. (2009). Remote Sensing with Imaging Radar.

[18] Hopper G.S., Campana S.B. (1993). Forward-looking Infrared Systems. The Infrared & Electro-Optical Systems Handbook, Passive Electro-Optical Systems.

[19] Carrara W.G., Goodman R.S., Majewski R.M. (1995). Spotlight Synthetic Aperture Radar.

[20] Northrop Grumman LN-100G Inertial Measurement Unit datasheet. http://www.northropgrumman.com/Capabilities/LN100GInertialNavigationSystem/Documents/ln100g.pdf.

[21] (1988). Air Data Computer. Technical Report TSO-C106. http://rgl.faa.gov/Regulatory_and_Guidance_Library/rgTSO.nsf/0/fdc3133eed60bdb986256dc600696543/$FILE/C106.pdf.

[22] PicoSAR. http://www.selex-es.com/it/-/picosar-1.

[23] ESGS GIS. http://egsc.usgs.gov/isb//pubs/gis_poster/.

[24] (2000). Digital Terrain Elevation Data (DTED): Performance Specification. https://dds.cr.usgs.gov/srtm/version2_1/Documentation/MIL-PDF-89020B.pdf.

[25] Hanssen R.F. (2001). Radar Interferometry: Data Interpretation and Error Analysis.

[26] Gatelli F., Monti Guarnieri A., Parizzi F., Pasquali P., Prati C., Rocca F. (1994). The wavenumber shift in SAR interferometry. IEEE Trans. Geosci. Remote Sens..

[27] Dall J. (2007). InSAR Elevation Bias Caused by Penetration into Uniform Volumes. IEEE Trans. Geosci. Remote Sens..

[28] Ghiglia D.C., Pritt M.D. (1998). Two Differential Phase Unwrapping: Theory, Algorithms and Software.

[29] Lingua A., Marenchino D., Nex F. (2009). Performance Analysis of the SIFT Operator for Automatic Feature Extraction and Matching in Photogrammetric Applications. Sensors.

[30] Heady B., Kroenung G., Rodarmel C. High resolution elevation data (HRE) specification overview. Proceedings of ASPRS/MAPPS Conference.

[31] Rizzoli P., Bräutigam B., Kraus T., Martone M., Krieger G. (2012). Relative height error analysis of TanDEM-X elevation data. ISPRS J. Photogramm. Remote Sens..

